# Web-Based Intervention to Act for Weight Loss in Adults With Type 2 Diabetes With Obesity (Chance2Act): Protocol for a Nonrandomized Controlled Trial

**DOI:** 10.2196/48313

**Published:** 2024-01-31

**Authors:** Noraini Mohd Saad, Mariam Mohamad, Aimi Nadira Mat Ruzlin

**Affiliations:** 1 Faculty of Medicine Universiti Teknologi MARA Sungai Buloh, Selangor Malaysia

**Keywords:** readiness to change, behavior change, diabetes, overweight, weight reduction, eHealth, obesity

## Abstract

**Background:**

In adults with type 2 diabetes (T2D), weight loss can improve hemoglobin A_1c_, blood pressure, and triglycerides, and reduce the frequency of medications needed. Unfortunately, a large proportion of these individuals are not ready to initiate weight efforts, making existing obesity management strategies less effective. Many digital health interventions aim at weight loss, but there is still limited evidence on their effectiveness in changing weight loss behavior, especially in adults with T2D.

**Objective:**

This study aims to develop and validate “Chance2Act,” a new web-based intervention, designed specifically to facilitate behavioral change in adults with T2D with obesity who are not ready to act toward weight loss. Then, the effectiveness of the newly developed intervention will be determined from a nonrandomized controlled trial.

**Methods:**

A web-based intervention will be developed based on the Transtheoretical Model targeting adults with T2D with obesity who are not ready to change for weight loss. Phase 1 will involve the development and validation of the web-based health intervention module. In phase 2, a nonrandomized controlled trial will be conducted in 2 government health clinics selected by the investigator. This is an unblinded study with a parallel assignment (ie, intervention vs control [usual care] with an allocation ratio of 1:1). A total of 124 study participants will be recruited, of which 62 participants will receive the Chance2Act intervention in addition to the usual care. The primary outcome is the changes in an individual’s readiness from a stage of not being ready to change (precontemplation, contemplation, or preparation stage) to being ready for weight loss (action stage). The secondary outcomes include changes in self-efficacy, decisional balance, family support for weight loss, BMI, waist circumference, and body fat composition.

**Results:**

The phase 1 study will reveal the intervention’s validity through the Content Validity Index and Face Validity Index, considering it valid if both indices exceed 0.83. The effectiveness of the intervention will be determined in phase 2, where the differences within and between groups will be analyzed in terms of the improvement of stages of change and all secondary outcomes as defined in the methodology. Data analysis for phase 2 will commence in 2024, with the anticipated publication of results in March 2024.

**Conclusions:**

If proven effective, the result of the study may give valuable insights into the effective behavioral modification strategies for a web-based intervention targeting adults with T2D with obesity but not yet ready to change for weight loss. This intervention may be replicated or adopted in different settings, focusing on behavioral modification support that patients need. This study offers a deeper understanding of the application of behavior change techniques for a more holistic approach to obesity care in T2D.

**Trial Registration:**

ClinicalTrials.gov NCT05736536; https://clinicaltrials.gov/study/NCT05736536

**International Registered Report Identifier (IRRID):**

DERR1-10.2196/48313

## Introduction

Obesity has become one of the leading global health concerns over the last century. Numerous studies have proved the complex relationship between obesity and type 2 diabetes (T2D). The majority of patients with T2D are either overweight or obese, thus increasing their long-term risks of developing cardiovascular diseases and diabetes complications [1]. The World Health Organization (WHO) has reported that around 2.8 million people die each year as a result of being overweight or obese [2]. In patients with T2D, weight loss can improve hemoglobin A_1c_, blood pressure, triglycerides, and health-related quality of life, and reduce the frequency of medications required [3]. A significant reduction of body weight (>10%) can induce diabetes remission even among those with advanced disease and established diabetes complications [4,5].

In Malaysia, the prevalence of obesity among patients with T2D has been increasing each year, whereby 84% of the patients with T2D were recorded to be either overweight or obese in 2019 [6]. Unfortunately, studies have shown that a large proportion of them (59%-79%) are still not ready to act on losing weight [7,8]. Since they do not have the intention of losing weight, they would not be interested in practicing or acting upon the recommendations given by the primary care personnel and thus, are deprived of the benefit of losing weight.

Interventions in behavior modification have been proven as an important component in a successful weight control program, in addition to the dietary and physical activity components. A European guideline has shown that behavioral change can induce 5%-15% weight loss among overweight or obese individuals [9]. Theories on behavior change should be applied when developing any health intervention because it increases the likelihood of being more effective. Among the theories, the Transtheoretical Model (TTM) uniquely describes the behavior change process from not being ready (precontemplation, contemplation, and preparation) to the active stage (action and maintenance). Applying TTM may help researchers in understanding the behavioral issue and help to develop a tailored intervention based on the individualized readiness stage [10]. A systematic review has found that by implementing the stages of change of TTM, there were improvements in physical activity and dietary habits, specifically in terms of exercise duration and frequency, reduction in dietary fat intake, as well as increment in fruits and vegetable consumption [11]. Unfortunately, the current management of T2D does not identify those who are ready or not ready to make changes in their daily life [12]. Clearly, there is a need to help the large proportion of patients who are not ready to change to start taking action for weight loss.

Intervention in behavior modification should address the role of social environment on an individual’s readiness to lose weight. Weight loss-related behaviors, specifically healthy diet and physical activity are embedded in the social context, particularly the family context. Therefore, addressing behavior modification embedded in daily family life might be a promising approach to boost family support, thereby facilitating progress. Family support can be received in the form of companionship, emotions, information sharing, and instrumental assistance to facilitate the weight loss journey [13]. Integration with the family support component can lead to a more maintainable behavioral change, a better improvement of diabetes outcomes, as well as help to improve the health of family members [14,15].

Over the decades, web-based health technologies are increasingly used as a delivery mode for health promotion and prevention. The web-based platform will provide a good opportunity to improve the management of patients with T2D with obesity. Implementing web-based health promotion and prevention can widen the opportunity to reach specific target groups, lower the cost of implementation, and improve the health of the population [16]. It may overcome problems such as time constraints, privacy concerns, and the negative perception of being obese. Furthermore, web-based intervention can be delivered within a social system so that all members, for example, of a family can simultaneously and collectively take part in the obesity intervention and share their plans, goals, and progress [17]. To increase the efficiency of the interventions, the intervention can be coupled with tailored feedback and counseling [18]. Unfortunately, there is limited knowledge in the literature on web-based health intervention that has been developed based on the TTM targeting adults with T2D with obesity and integrating the family support component.

The objective of this trial is to develop and validate “Chance2Act,” an innovative web-based intervention grounded in the TTM, designed specifically to facilitate behavioral change in overweight or obese adults with T2D who are not ready to take action toward weight loss. Then, the effectiveness of the newly developed digital health intervention will be determined based on a nonrandomized controlled trial.

## Methods

### Overview

This study will be conducted in two phases, which are (1) phase 1: the development and validation of the Chance2Act intervention module and (2) phase 2: the nonrandomized controlled trial of the Chance2Act intervention.

### Phase 1: Content Development and Validation

Phase 1 of the study concerns the development and validation of the health intervention module. To get the initial ideas, framework, and justification for the selection of topics, a literature review and document analysis will be done. The content selection will be based on the key findings from the review of previous studies, the available local and international guidelines, as well as published reports, and available statistical data. The search sources will involve web-based databases, websites and printed materials (guidelines, pamphlets, posters, factsheets, reports, etc) that incorporate behavior change theories and family support components.

In addition, experts in related fields from different sectors (government, private, and nongovernmental organizations) will be invited for informal interview sessions to share experiences and give input for the new intervention. Further exploration will be conducted through informal interviews with overweight or obese adults with T2D, focusing on the knowledge, awareness, and practice of unhealthy behaviors, challenges, and facilitators for weight loss.

The contents of the health education module will be arranged according to the Process of Change construct of TTM. Then, the contents of the module will be drafted including the texts and graphics, as well as the formatting and layout for the website which acts as the medium of delivery for the health intervention. The local language (Malay language) will be used for the communication. The website will be arranged with consideration of the appropriate sequence. An attractive illustration will be used to ensure participants’ engagement and hence complete the entire package of the intervention module.

For the validation of the intervention module, 6 experts from relevant fields related to obesity and diabetes management will be selected to evaluate the newly developed health intervention module. The experts will consist of (1) an epidemiologist of noncommunicable diseases, (2) a health education expert, (3) a family medicine specialist, (4) a dietician, (5) a sports medicine specialist, and (6) a medical officer in-charge of the diabetes program in a health clinic. The Content Validity Index (CVI) will be analyzed to determine the relevance of the intervention content.

**Figure 1:**
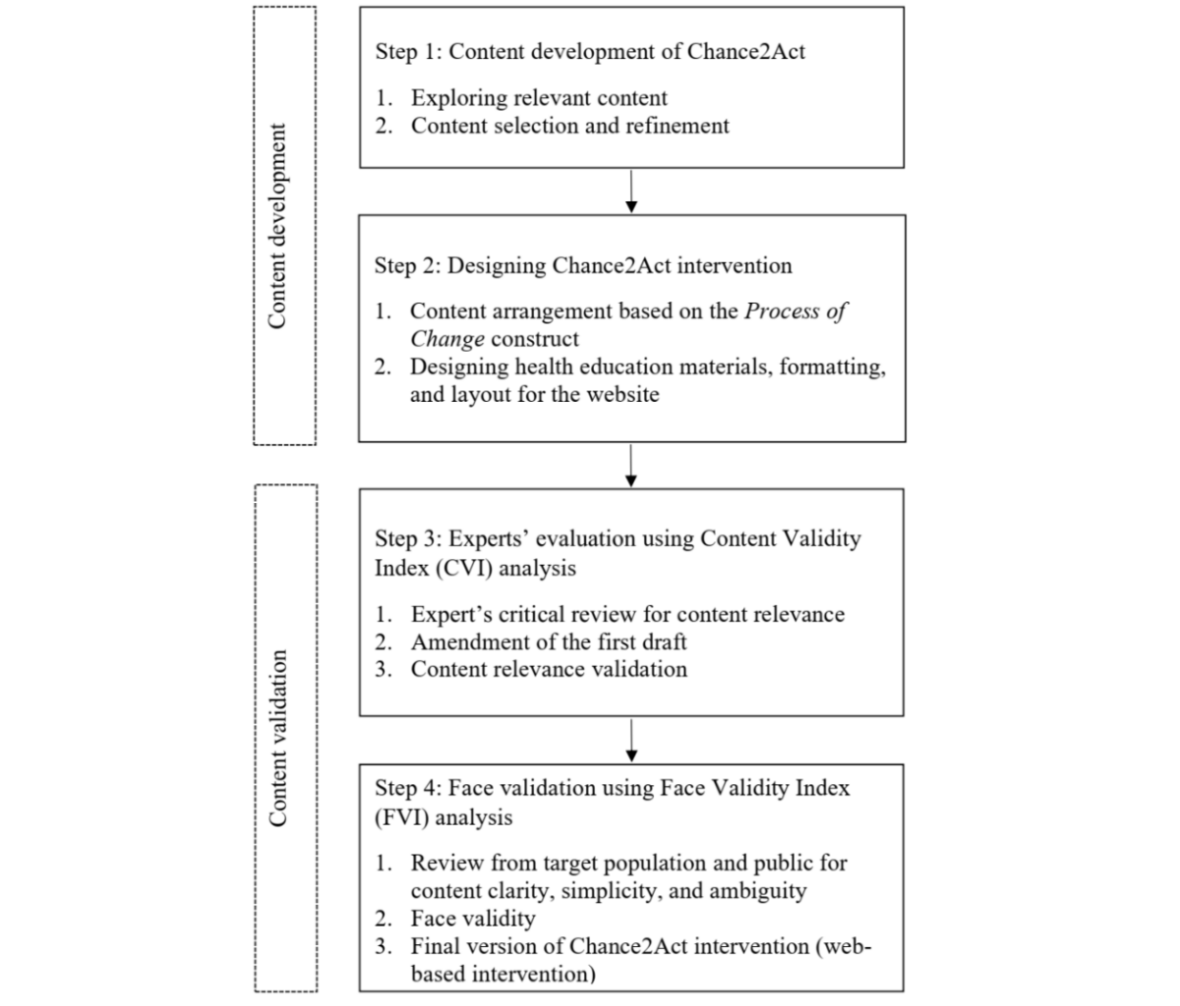
Study flowchart for phase 1.

A face validation study will be conducted among overweight or obese adults with T2D and the public to determine the clarity, simplicity, and ambiguity of the intervention content. It will be quantified by using the Face Validity Index (FVI). After being proven to be valid to achieve the primary objective, the final version of Chance2Act Health Intervention will be ready to be used in phase 2: the nonrandomized controlled trial. Figure 1 illustrates the study flowchart for phase 1.

### Phase 2: Nonrandomized Controlled Trial

#### Study Setting

**Figure 2:**
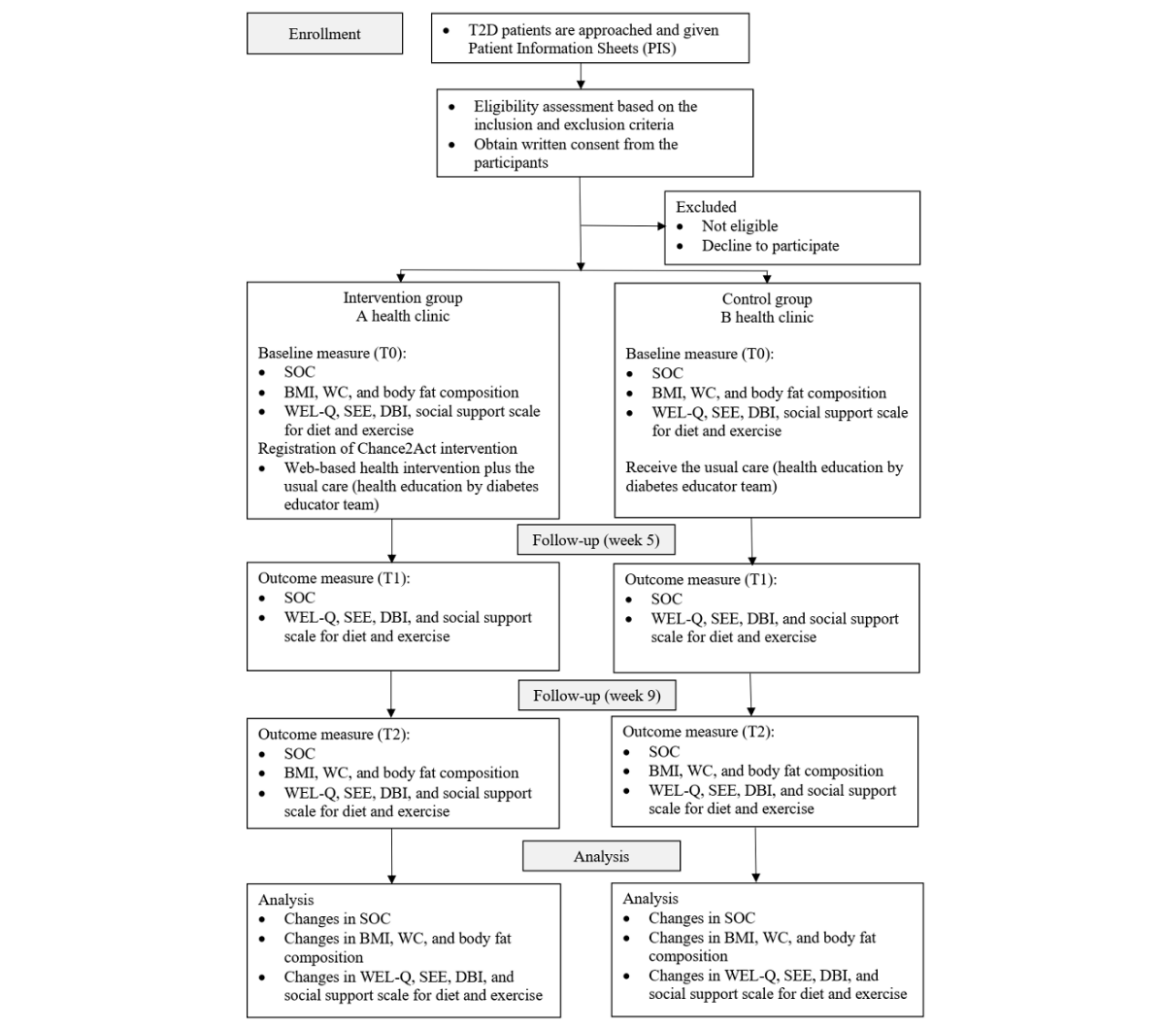
Study flowchart for phase 2. DBI: Decisional Balance Inventory; SEE: Self-efficacy for Exercise; SOC: Stages of Change; T2D: type 2 diabetes; WC: waist circumference; WEL-Q: Weight-efficacy Lifestyle Questionnaire.

Phase 2 of the study will be conducted as a nonrandomized controlled trial, which is aimed to determine the effectiveness of the Chance2Act intervention. The study is expected to be conducted over 4 to 6 months, at 2 government health clinics in Pahang state, Malaysia. These 2 health clinics will be enrolled by the researcher and are expected to have similarities in terms of the sociodemographic background of the local community. The assignment into either the intervention or control group will be based on the health clinic where the participants are getting their treatment from. One health clinic will be assigned as the intervention arm whereas another clinic as the control arm. The study flowchart for phase 2 is summarized in Figure 2. This is an unblinded study with a parallel assignment (ie, intervention vs control [usual care] with an allocation ratio of 1:1). Blinding is not possible due to the nature of the intervention which is mainly health education.

To avoid contamination, the 2 health clinics will not be selected from neighboring localities. Access to the Chance2Act website will be restricted only to the registered participants and family members from the intervention arm. A username and password will be provided to log in to the website. The participants, family members, and the medical staff at the participating health clinics are not allowed to share any intervention material with a third party.

#### Study Population

The target population is patients with T2D aged 20-60 years old, with a BMI of more or equal to 23 kg/m^2^, who are not ready to act for weight loss. Study participants will be selected via purposive sampling method to ensure homogeneity between the 2 arms.

#### Participants Recruitment

The patients with T2D, who come for a routine appointment at the selected health clinics during the recruitment period will be approached, given the Patient Information Sheet (PIS) about the study, and invited to participate. If they are willing to participate, they will be screened by the investigators to determine their eligibility based on the inclusion and exclusion criteria. Then, written informed consent will be obtained from those who are eligible. Participants from the intervention arm will be given a username and password to access the Chance2Act intervention, whereas participants from the control arm will continue the existing health education module in the health clinic.

#### Eligibility Criteria

The patients with T2D aged 20 to 65 years old who fulfill all the following inclusion criteria will be included (1) BMI of more or equal to 23kg/m^2^; (2) in the precontemplation, contemplation, or preparation stage of change based on the s-weight questionnaire [19]; (3) able to understand, read, and write in the Malay language; (4) have access to any electronic device with internet service; (5) able to understand information and perform tasks in the digital environment, especially website; and (6) have at least 1 adult family member who is living in the same household.

Patients who fulfill any of the following criteria will be excluded (1) patients who are pregnant; (2) patients with advanced comorbidity and mental conditions that will affect participation and understanding of the study protocol, for example, vision problem, physically unfit to stand up and walk, advanced heart failure or cancer, and schizophrenia; and (3) patients who die or are transferred out to other health clinics during the study period.

#### Sample Size Calculation

The sample size was calculated using OpenEpi (version 3; Kelsey et al) open-source calculator for cross-sectional, cohort, and randomized control trials. To obtain 95% CI and 80% power of the study (α=.05), the minimum sample size required in each arm is 44 to achieve a 30% proportion difference postintervention [20]. Considering a 40% (n=21 participants) attrition rate, we aim for a total of 65 participants per arm.

#### Chance2Act Health Intervention

The Chance2Act digital health intervention is a web-based behavior modification program based on the 4 main constructs of the TTM. This model uses the stages of change to integrate the principles and processes of behavior change. The stages of change are influenced by the processes of change (activities people use to progress through stages), decisional balance (an individual’s relative weighing of the pros and cons of changing), and self-efficacy (situation-specific confidence that people can cope with without relapsing to their former behavior) [21]. This intervention emphasizes the role of the immediate family members in creating a healthy social environment favoring weight loss. The main components of the intervention will be identified from an extensive review of published studies, health education materials, and gray literature that can boost the readiness to change for weight loss. This is the first version of the study protocol.

#### Delivery of the Chance2Act Intervention

The Chance2Act intervention will be delivered to the individual participants by the investigators. Participants will be given the Chance2Act website using their login account and password. Access to the website is free of charge. The intervention will comprise 4 main modules and will be delivered in sequence. Each module will take approximately 2 weeks to be completed. The sequence of the intervention was developed based on the Process of Change construct of the TTM [21].

The baseline measurements will be taken before the initiation of the intervention. Follow-up will be done by the investigator after the completion of the first 2 modules, approximately at week 5, and after the completion of the whole package of the intervention, at week 9. In addition, participants can seek help from the investigators to navigate through the functions of the Chance2Act website through the built-in messaging system.

The Chance2Act is a tailored intervention whereby the participants will receive personalized coaching about their weight-related behavioral problems to boost their motivation via the built-in messaging system. The principal investigator will actively monitor the progress of each participant and intervene according to the health education module. Information will be given through text on the website, sets of infographics, animations, and videos. Health quizzes will be conducted to assess the comprehensibility of the module. They will be given printed materials such as weight loss checklists and dietary and exercise weekly plan templates to assist their weight loss journey. Each participant will have at least 1 adult family member to join as part of the intervention modules. The family members will be guided on the ideal way to fully support the participants’ efforts for weight loss. They will receive clear information on their role during the weight loss journey.

Participants are expected to finish each module in 2 weeks and give the required feedback and responses at the end of the module accordingly. Follow-up will be done by the investigators. Participants who did not comply with the follow-up requirements will be considered a loss to follow-up. Data analysis will be by the intention-to-treat (ITT). There is no specific concomitant care and intervention that are permitted or prohibited during the trial.

#### Data Collection

For both arms, the data for phase 2 will be obtained from a set case report form (CRF) which consists of demographic data, self-administered questionnaires, anthropometric measurements, and disease profiles. At week 0 (T0), once consented, anthropometric measurements will be taken by the investigator and clinical data will be obtained from the medical records.

Clear written and verbal instructions will be given on how to answer the questionnaires. Participants will be requested to circle which options suit them the most. Participants will be allowed to answer the questionnaires while waiting their turn for medical consultation. They will be encouraged to seek clarification from investigators at any time, should any queries arise.

Before the initiation of the study, all investigators will be trained regarding the study procedures to minimize variability and bias during the data collection. The study procedures will comply with the Malaysian Guideline for Good Clinical Practice, fourth edition [22].

#### Study Instruments

For the anthropometric measurement, the height will be taken using a wall mounted stadiometer (SECA 206) and recorded to the nearest 0.1 cm. The body weight and body fat composition (total fat percentage and visceral fat percentage) will be measured using a body fat composition monitor (Omron HBF-222T) with an accuracy of 0.1 kg and 0.1%, respectively. Waist circumference will be measured to the nearest 0.2 cm using nonstretchable measuring tape midway between the lower margin of the 12th rib and the top of the iliac crest, just above the umbilicus in a horizontal plane.

The set of self-administered questionnaires will consist of (1) a Stages of Change (SOC) questionnaire using the S-weight; (2) a Weight-efficacy Lifestyle Questionnaire (WEL-Q); (3) Self-efficacy for Exercise (SEE); (4) a Decisional Balance Inventory (DBI) for weight loss; and (5) social support scale for diet and exercise. Verbal instruction will be given on how to complete the questionnaire. On average, participants will be expected to spend approximately 30 minutes to complete the questionnaires. Once they have finished, they will submit the questionnaires to the investigator to ensure the completeness of the responses given.

The S-weight questionnaire was initially developed in English and Spanish language simultaneously [19,23]. This questionnaire has been translated into Malay language and tested among the adolescent obese population [24]. It consists of a brief series of self-report questions assessing weight loss intentions and current activities. The S-weight has 5 mutually exclusive items each representing the 5 stages of change: precontemplation, contemplation, preparation, action, and maintenance. S-weight has been reported as an efficient tool to assess the readiness to change in weight management by a review [25].

A validated Malay version of WEL-Q will be used to assess dietary self-efficacy for weight loss. It is used to measure an individual’s confidence to control eating in specific circumstances. It consists of 20 items representing 5 subscales which are negative emotions, availability, social pressure, physical discomfort, and positive activities. WEL-Q uses a Likert scale ranging from 0 (not confident) to 9 (very confident). The total score of each subscale is ranged between 0 and 36. The higher score shows better control to resist eating. WEL-Q has been tested among the Malaysian population with T2D. It is reported to have a good internal consistency reliability with Cronbach α of .893. Construct validity was measured by the item total correlation of *r*>0.700 and *P*<.01, and interitem correlation of *r*<0.005 and *P*<.01 [26].

The SEE was originally developed by Bandura and has been modified to suit the Malaysian population. It consists of 18 items measuring an individual’s confidence to maintain routine exercises. A 5-point Likert scale is used ranging from 1 (cannot do) to 5 (certain can do). The Malay version of SEE has been tested among Malaysian patients with T2D with a composite reliability (CR) of 0.921 [27].

The DBI was developed in the English language by O’Connell and Velicer in 1988 [28]. It measures an individual’s belief of the perceived pros and cons of weight loss. It consists of 20 Likert scale items, 10 items for pro (even-numbered items) and 10 items for cons (odd-numbered items). The scale ranges from 1 (not important at all) to 5 (very important). The total score range is 10 to 50. The Malay version of DBI shows good reliability. For the pro scale reliability was reported as Cronbach α=.902, intraclass correlation (ICC) (95% CI) is 0.88 (0.86-0.90). The con scale Cronbach α=.739 and ICC (95% CI) is 0.72 (0.70-0.78) [24].

The social support scale for diet and exercise was developed in the English language in 1987 [29]. In this study, the social support for diet and exercise will be measured separately and used specifically to measure the perceived social support received from the family members. The social support scale for diet consists of 10 items. Items 1-5 measure the encouragement, whereas items 6-10 measure discouragement received from the family members. The social support scale for exercise behavior consists of 12 items. The measurement uses a Likert scale ranging from 1 (never) to 5 (very often). A higher score implies that the participants have better social support received from their family members to lose weight. These questionnaires have been translated into Malay language and validated. This questionnaire has been proven valid and reliable. The social support for diet has internal consistency reliability of Cronbach α of .61-.91 and test-retest reliability, *r* of 0.55-0.86 [29]. The social support scale for exercise was reported to be reliable for the Malaysian population. It was reported to have a CR of 0.918, an average variance extracted of 0.560, and an ICC (based on test-retest) of 0.920 [30].

#### Study Outcome

Outcome measures are categorized into primary and secondary outcomes. These measures will be obtained from both the intervention and control arm at baseline, fifth week, and ninth week after the initiation of the intervention.

The primary outcome will be measured by the changes of the stages of change using the Malay version of the S-weight questionnaire. The secondary outcomes will be measured by (1) a change in dietary self-efficacy will be measured by the WEL-Q, (2) a change in exercise self-efficacy will be measured by the SEE, (3) a change in decisional balance for weight loss will be measured by the DBI, (4) change in family support for diet and exercise will be measured by the social support scale for diet and exercise, (5) change in BMI, (6) change in waist circumference, and (7) change in body fat percentage.

#### Control Group

The control group will continue to receive the usual care at the health clinic. The usual care includes the health talk and individual or group counseling sessions with the diabetes educators, physiotherapists, dieticians, or medical officers as scheduled by the health clinic. Participants from the control group will be offered to participate in the intervention after the completion of T2 data collection.

### Data Management and Statistical Analysis

For phase 1, the data will be entered in Microsoft Excel (Microsoft Office 365) and ready for analysis. The content validity will be assessed by the experts’ ratings. The CVI will be assessed based on 2 indices, the Item-level Content Validity Index (I-CVI) and Scale-level Content Validity Index (S-CVI). The criteria that will be assessed are mainly content relevance. The I-CVI will reflect each item or intervention activity relevancy, whereas S-CVI will reflect the overall module relevancy. Then, the face validation will be determined based on the FVI. Similarly, the FVI will be assessed based on 2 indices, the Item-level Face Validity Index (I-FVI) and the Scale-level Face Validity Index (S-FVI). The criteria that will be evaluated are the module clarity, simplicity, and ambiguity. For both CVI and FVI, the results of at least 0.83 will be considered acceptable [31,32].

For phase 2, the data will be entered into IBM SPSS Statistics (version 27; IBM Corp). Each CRF will be given a unique identifier. Screening for missing, redundant data or wrong coding will be done. The investigator will try to contact the participants to complete the missing data via telephone. Then, any remaining missing data will be dealt with an appropriate missing data handling method. For redundant data, only 1 data will be selected. The wrongly coded data will be corrected accordingly based on the CRF. Then, the data will be ready for analysis.

For the descriptive statistics, the normality tests will be used to determine the distribution of the data. The normally distributed will be described by using mean and SD, whereas not normally distributed data will be described by using the median and IQR. The categorical data will be presented by using frequency and percentage.

To determine the homogeneity between the intervention and control group, an independent *t* test and chi-square test will be used to compare the demographic characteristics of the participants. The ITT analysis will be applied to measure the effectiveness of the Chance2Act intervention on the primary and secondary outcomes. The analysis models are within-group differences and between-group differences. The Generalized Estimating Equations (GEE) will be used as statistical analysis. A *P* value of less than .05 is considered statistically significant with 95% CI. Interim analysis will be conducted at T1 which is estimated at week 5. However, only certain outcome measures will be analyzed as predetermined in the phase 2 study flowchart.

### Ethical Considerations

This study has been approved by the Medical Research and Ethics Committee, Ministry of Health Malaysia (NMRR ID-22-01073-4YT IIR) and Universiti Teknologi MARA Research Ethics Committee (REC/07/2022PG/MR/157). The ethics committees will be informed in case of any changes to the study protocol. Before participants’ enrollment, PIS will be given and explained to all the participants. Written informed consent will be obtained once patients understand the study well. The research assistants will be trained to conduct these procedures. The risk of privacy breaches is minimal. The website will only contain health education material. None of the personal information and medical information will be uploaded on the website. The data for the outcome measures will be stored by the investigator in a password-protected computer and cloud account provided by the Ministry of Health Malaysia (MyGovucUC 2.0 account). The data will be destroyed after 3 years postcompletion of the study. The permission to use the questionnaires has been obtained from the authors with proper citation.

## Results

The objective of the phase 1 study is to develop and validate a new web-based intervention (Chance2Act) to facilitate behavioral change for weight loss among adults with T2D with obesity. To confirm the accomplishment of the study objectives, the data gathered from the expert panel’s rating and the panel of raters will be analyzed using the CVI and the FVI. The phase 1 study was initiated in October 2022 and is expected to be completed within an 8-month period. Until May 2023, the analysis of the CVI data for the intervention has been finalized and is currently undergoing evaluation by the panel raters for FVI, marking the conclusion of phase 1.

After the validity of the intervention has been confirmed, the effectiveness of the intervention will be determined through a nonrandomized controlled trial. The data collection is expected to have started on June 2023 and to be completed within 4 to 6 months. The analysis will allow us to compare results between the intervention and the control arms, as well as changes within the arm itself in terms of the improvement of the stages of change for weight loss as per the TTM. We also hypothesize that the intervention can result in improvement in self-efficacy, decisional balance, and family support which ultimately may contribute to weight loss. Data analysis for phase 2 will commence in 2024, with anticipated publication of results as early as March 2024. This study was funded in July 2022.

## Discussion

### Principal Findings

This trial will determine the validity and effectiveness of Chance2Act, a newly developed web-based intervention in changing the behavior of adults with T2D with obesity to initiate weight loss efforts. The validity of the intervention is determined in phase 1 using the CVI and FVI. These tools are widely recognized as quantitative methods to evaluate the content validity of health intervention research [31,32]. In the context of developing interventions, CVI is essential to evaluate whether the elements of the intervention are relevant in relation to the initial objectives or the intended outcomes [33]. Likewise, face validity is crucial to ensure the target users’ understanding of each item in the intervention. An intervention that appears relevant, clear, and comprehensible to the participants is more likely to be effective [34]. Integrating both CVI and FVI in the development phase ensures that the intervention is not only theoretically sound but also remains understandable and clear to the target audience.

Then, the results of phase 2 will determine the effectiveness of the intervention in changing the behavior of adults with T2D with obesity toward weight loss. The analysis will focus on the differences of all outcome measures to demonstrate the effects of the intervention both within and across the different groups. If the hypothesis is confirmed, the findings of this study will provide valuable insights into effective strategies for web-based intervention aimed at behavior modification in the context of weight management for adults with T2D.

### Strengths and Limitations

To our best knowledge, the Chance2Act intervention is the first web-based intervention that promotes behavior change, specifically designed for adults with T2D with obesity but not yet prepared to start losing weight. Developing an intervention for this population group is crucial, as a significant number (59%-79%) of them are not yet actively making weight loss efforts [7,8]. We hope that this intervention can contribute to better outcomes of obesity care in patients with T2D, thus, potentially leading to the metabolic benefits associated with weight loss.

In terms of the study design, the selection of a nonrandomized controlled trial method is acceptable due to the nature of the intervention given. Since the health interventions are delivered through a website, blinding of the study participants and researcher is not possible. The participants need to be aware of group assignments, thereby they will access the website and be involved in the intervention.

Another potential limitation that may arise during the study is that participants who do manifest interest are limited to patients with T2D with good digital literacy skills only. As a result, the findings of the study may not be applicable to a broader population, especially those with limited digital literacy. Digital literacy of the study participants may influence the conclusions concerning the uptake and attrition rate of the intervention.

### Conclusions

To conclude, the potential findings from this study may bring significant implications of the effective strategies for a web-based intervention aimed at adults with T2D with obesity, who are not actively taking action for weight loss. If successful, the newly developed and validated intervention may be replicated or adapted in different settings, addressing the behavior change resistance for weight loss in adults with T2D with obesity. We hope that the Chance2Act intervention will benefit the target group in achieving better diabetes and metabolic control through effective weight loss. These findings may give valuable insights to inform future public health strategies toward a broader approach to obesity management in T2D, emphasizing the importance of behavioral modification in achieving maintainable weight reduction.

## Abbreviations

CR: composite reliability
CRF: case report form
CVI: Content Validity Index
DBI: Decisional Balance Inventory
FVI: Face Validity Index
GEE: Generalized Estimating Equations
I-CVI: Item-level Content Validity Index
I-FVI: Item-level Face Validity Index
ICC: intraclass correlation
PIS: Patient Information Sheet
S-CVI: Scale-level Content Validity Index
S-FVI: Scale-level Face Validity Index
SEE: Self-efficacy for Exercise
SOC: Stages of Change
TTM: Transtheoretical Model
T2D: type 2 diabetes
UiTM: Universiti Teknologi MARA
WEL-Q: Weight-efficacy Lifestyle Questionnaire
WHO: World Health Organization

## Data Availability

The data sets generated or analyzed during this study are not publicly available in order to comply with the ethical board requirements and participants’ confidentiality protection but are available from the corresponding author upon reasonable request, who will then seek necessary permissions from the Director General of Health Malaysia.
